# Measuring PARP1 mobility at DNA damage sites by segmented fluorescence correlation spectroscopy

**DOI:** 10.1016/j.bpj.2025.05.013

**Published:** 2025-05-16

**Authors:** Elisa Longo, Greta Paternò, Alberto Diaspro, Luca Lanzanò

**Affiliations:** 1 Department of Physics and Astronomy “Ettore Majorana”, University of Catania, Catania, Italy; 2 Nanoscopy, CHT Erzelli, Istituto Italiano di Tecnologia, Genoa, Italy; 3 DIFILAB, Department of Physics, University of Genoa, Genoa, Italy; 4 Istituto Nazionale di Fisica Nucleare (INFN), Sezione di Catania, Catania, Italy; 5 Centro Siciliano di Fisica Nucleare e Struttura della Materia (CSFNSM), Catania, Italy

## Abstract

Segmented fluorescence correlation spectroscopy (FCS) improves the accuracy of FCS measurements in cells by analyzing data in short temporal segments. We have recently demonstrated the possibility of performing segmented FCS using a commercial confocal laser scanning microscope, enabling the measurement of molecular diffusion in different subcellular regions. In this study, we apply segmented FCS to investigate the dynamics of poly(ADP-ribose) polymerase 1 (PARP1), a protein that plays a central role in the DNA damage response. We perform fast line scanning across the nucleoplasm of live cells to measure the recruitment kinetics of PARP1 at DNA damage sites after laser micro-irradiation. The segmentation of FCS data allows us to distinguish between the damaged and undamaged regions and to measure the mobility of PARP1 in the two regions. We find reduced mobility of PARP1 at DNA damage sites, described as the appearance of a binding fraction, whereas the diffusion of PARP1 is unaltered outside the DNA damage region. Additionally, we investigate the effect of photobleaching on the measured dynamics.

## Significance

Poly(ADP-ribose) polymerase 1 (PARP1) is a protein involved in the DNA damage response. Understanding the dynamics of PARP1 at DNA damage sites can provide important insights into its functional role in the cellular response to DNA damage. Here, we apply segmented fluorescence correlation spectroscopy to measure the mobility of PARP1 after laser-induced DNA damage. This approach allows us to quantify PARP1 mobility in damaged versus undamaged chromatin regions. We find reduced mobility of PARP1 in the region of DNA damage, whereas the diffusion is unaltered outside the DNA damage region. In addition, we discuss the impact of photobleaching on the measured dynamics.

## Introduction

Cells are exposed daily to factors that cause DNA damage of different types, such as UV light, environmental exposure, and chemical agents, which can induce DNA lesions such as single- and double-strand breaks. There are several mechanisms of response to DNA damage mediated by protein-protein interactions that are essential for the specific recruitment of DNA damage response factors to the sites of DNA damage. Gibson et al. (1) summarized the processes concerning the dynamics of DNA damage repair, highlighting how poly(ADP-ribose) (PAR) polymerases (PARPs) control many cellular processes. In particular, PARP1 is a multifunctional enzyme that detects DNA strand breaks and signals the presence of DNA damage to other repair proteins through a process called PARylation, which facilitates the recruitment of DNA repair complexes at the sites of damage. When PARP1 binds to DNA strand breaks, it signals the induction of repair processes by activating its enzymatic activity, which is useful for the recruitment of many DNA repair proteins (2). In addition to its involvement in DNA repair, the highly abundant PARP1 also plays a key role in regulating chromatin structure and transcription. Indeed, by using PAR chains, PARP1 binds chromatin with high affinity, facilitating chromatin relaxation to allow DNA repair processes to proceed efficiently.

The recruitment dynamics of proteins to DNA damage sites have been studied using various advanced live-cell imaging techniques (3). One widely employed method is fluorescence recovery after photobleaching (FRAP), which involves the localized perturbation of fluorescence in cells expressing a protein of interest, tagged with a specific fluorophore (4,5). FRAP can distinguish between different populations of molecules based on their stability in associating with DNA lesions (6,7). Another frequently used technique is fluorescence correlation spectroscopy (FCS), which provides quantitative data from the cellular environment (8,9). FCS is a noninvasive method that analyzes the spontaneous fluctuations of fluorescence intensity in a defined observation volume (10,11). These fluctuations are characterized by an autocorrelation function (ACF) whose width depends on the diffusion coefficient of the particles (12). More advanced FCS analysis allows the detection of so-called diffusion laws and the accurate measurement of diffusion and binding dynamics (13,14). The FCS method allows for the extraction of key parameters involved in protein dynamics at sites of damage, such as diffusion coefficients and association/dissociation rates (15). Compared to FRAP, FCS provides a more refined characterization of the diffusive components and can detect rapid recruitment events with greater precision (6). Recently, the pair correlation function technique has been employed to characterize the turnover of repair factors at DNA damage sites. The pair correlation function is based on the analysis of fluorescence fluctuations along a single confocal scan line (16,17). By cross correlating the signals from two distinct pixels, the characteristic transit time of a molecule can be determined, offering valuable insights into the dynamics of protein recruitment at damaged DNA sites (18).

FRAP has been crucial in clarifying the dynamics of PARP1 interactions with substrates and their accumulation at DNA repair foci, including the effect of PARP inhibitors (19,20). Recently, single-molecule imaging revealed that PARP1 molecules rapidly exchange at DNA damage sites (21). Karpinska et al. used FCS to quantify the intracellular distribution of a fluorescent olaparib analog (22). Kozlowski has shown that PARP1 is predominantly freely diffusing under normal conditions but transitions to chromatin-bound states upon induction of DNA damage (23).

Here, we describe a protocol for measuring the mobility of PARP1 at DNA damage sites by segmented FCS using a commercially available confocal laser scanning microscope. The approach is based on 1) the induction of DNA damage in Hoechst-sensitized cells by 405-nm laser micro-irradiation and 2) segmented line scanning FCS of PARP1 labeled with a chromobody tagged with RFP (PARP1-RFP). Segmented FCS is a variant of FCS where the raw data (i.e., intensity versus time) are divided and processed using short temporal segments to increase the accuracy of FCS measurements in live cells (24,25). We have recently shown how segmented FCS can be performed in a commercial microscope, using slow or fast line scan acquisition and segmented data processing with a user-friendly script in MATLAB (26). In this application, we perform a temporal segmentation of the line scanning data, useful 1) to eliminate artifacts due to photobleaching or movement of the DNA damage region and 2) to separate data segments corresponding to the DNA damaged region (identified by the accumulation of PARP1-RFP and thus a higher average intensity value) from data segments corresponding to nearby, nondamaged regions of the cell nucleus (identified by lower average intensity value).

We find that, outside the DNA damage region, the mobility of PARP1 is mainly characterized by a diffusive component, corresponding to the mobility of PARP1 in the chromatin environment. In contrast, inside the DNA damage region, we observe reduced mobility of PARP1, described by the occurrence of an additional binding component, indicating the interaction of PARP1 molecules with the site of DNA damage. We also investigate the impact of photobleaching on the measured dynamics.

## Materials and methods

### Schematic implementation of the method

**Figure 1:**
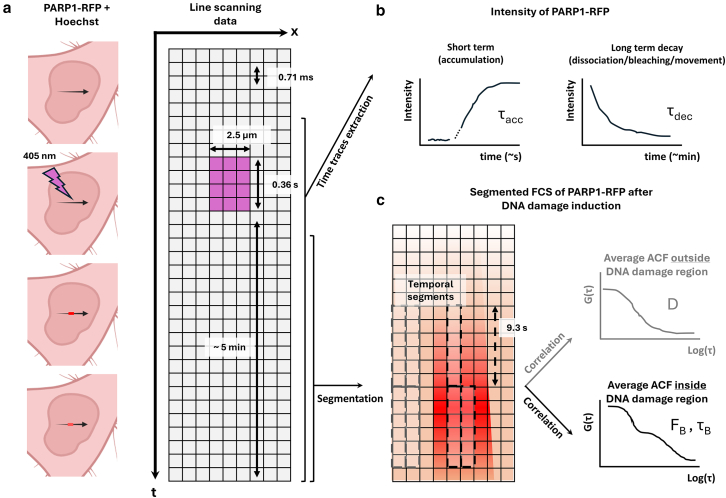
Schematic implementation of the method. (*a*) Line scanning data are acquired from a commercial laser scanning microscope and exported as images. DNA damage is induced by a 405-nm laser for 0.36 s at the center of the scanned line. (*b*) Intensity time trace analysis performed on data. Short-term profile analysis provides information about the accumulation time $\tau_{ACC}$ of PARP1-RFP in the region of damage in the seconds timescale. Long-term analysis, in the minutes timescale, gives us time of detachment from the damaged sites but may also be affected by movement of the DNA damage region and photobleaching. (*c*) Segmented FCS analysis: data are analyzed after the induction of DNA damage and segmented along the temporal axis of the carpet. Pixels of consecutive lines are correlated, and for each segment, the corresponding ACF is calculated. The segments are selected based upon the value of the reference intensity: segments with high PARP1-RFP intensity are assigned to the inside DNA damage region, whereas segments with low intensity are assigned to the outside DNA damage region. For each region, an average ACF is calculated and fitted.

Data acquisition is performed in a commercial laser scanning microscope (Fig. 1 *a*) by fast line scanning along the *X* axis in the nucleus of live cells labeled with a PARP1 chromobody tagged with TagRFP (PARP1-RFP) and Hoechst 33342. DNA damage is induced during line scanning by laser irradiation with a 405-nm laser (power measured at the sample: 0.3 mW). Irradiation was applied to a region of interest of 25 × 1 pixels, corresponding to a length of 2.5 *μ*m, for 512 consecutive lines, corresponding to a total irradiation time of 0.36 s. The accumulation of PARP1-RFP is then monitored with a 561-nm laser for 5 min (Fig. 1 *b*).

For the intensity analysis, we measured the time-dependent accumulation of PARP1-RFP in the DNA damage region (Fig. 1 *c*, *left*) and tracked the total intensity profile throughout the entire acquisition period (Fig. 1 *c*, *right*). For the segmented FCS analysis, we analyzed the data after induction of DNA damage (Fig. 1 *d*). We segmented all the data, and the fluctuation analysis was performed by correlating pixels of consecutive lines, with the temporal resolution determined by a line time (the inverse of the line scanning frequency) of 0.71 ms. The segment duration T is approximately 9.3 s unless specified otherwise. We selected segments outside and inside the DNA damage region (Fig. 1 *d*) based on intensity threshold, generating the corresponding average ACFs.

### Cell culture and labeling

HeLa cells (ATCC no. CCL-2, Manassas, Virginia) are cultured in Dulbecco’s modified Eagle’s medium (Gibco, 11965092, Waltham, Massachusetts) supplemented with 10% fetal bovine serum (Euroclone, ECS5000LH, Pero, Italy) and 1% penicillin/streptomycin (Life Technologies, 15140-122, Waltham, Massachusetts).

For fluorescence microscopy measurements, 20,000 cells/cm^2^ are seeded on 8-well Ibidi chambered coverslips and incubated at 37°C in 5% CO_2_ for 24 h. Cells are transiently transfected using the Lipofectamine 3000 transfection kit (Thermo Fisher Scientific, L3000-001, Waltham, Massachusetts) with a plasmid encoding for a PARP1 chromobody tagged with TagRFP (ChromoTek, xcr, Hauppauge, New York). After transfection, cells are incubated at 37°C in 5% CO_2_ for 24 h. Nuclear staining is performed by incubating the cells for 15 min at 37°C with a solution of Hoechst 33342 (Thermo Fisher Scientific; stock solution, 20 mM) in Dulbecco’s modified Eagle’s medium at a final concentration of 2 *μ*M. The cells are then replaced with complete medium.

### Data acquisition

All measurements are performed on a Leica TCS SP8 confocal laser scanning microscope using a 1.40 NA 63× objective (HCX PL APO CS2 63/1.40 Oil, Leica Microsystems, Wetzlar, Germany).

For PARP1-RFP, the excitation wavelength is set at 561 nm, with emission detection bands of 570–600 and 605–650 nm via hybrid detectors operating in photon counting mode. We used detection in two channels to cross correlate the signal and remove the detector noise. Data were acquired as XT (line scan) images in fast scanning mode by setting the scanner velocity at a line frequency of 1400 Hz (corresponding line time: 0.71 ms), obtaining an image format XT of 128 × 417. 690 pixels and a pixel size of 97 nm.

The measurements are performed with different 561-nm laser power settings: 0.3, 1.6, 14, and 68 *μ*W. The actual laser power is measured at the sample using an optical power and energy meter console (PM100D, Thorlabs, Newton, New Jersey).

### Data processing and analysis

The line scan (XT) data, saved as LIF files, are opened in ImageJ and then saved as TIF files. The TIF files are opened with a custom script in MATLAB (Mathworks, Natick, Massachusetts, https://github.com/llanzano/SegmentedFCS) and processed for segmentation and calculation of the ACF.

The data are segmented along the *Y* axis of the image (temporal axis) with a segment length set to 9.3 s so that each column is divided in 32 segments. Since we have 128 columns, the total number of segments is given by 128 × 32. For each segment (*i*,*j*), the software calculates the ACF given by

(1) $$G_{i,j}\left(\tau \right)=\frac{{\left\langle I_{i,j}\left(t\right)I_{i,j}\left(t+\tau \right)\right\rangle }_{T}}{{\left\langle I_{i,j}\left(t\right)\right\rangle }_{T}^{2}}-1,$$

where $I_{i,j}\left(t\right)$ is the fluorescence intensity of segment (*i*,*j*), *τ* represents the temporal lag, and the brackets indicate averaging over all the pixels of the segment. Since data are acquired by splitting the fluorescence signal in two channels, the ACF is calculated as the cross correlation of the two channels. The line time was calculated as 1/f, where f is the line scanning frequency set in the Leica SP8 software. The average value of the intensity calculated in each segment ${<I}_{i,j}\left(t\right)>$ is shown on a 128 × 32 map and used to select the segments. The segment intensity value is normalized to the maximum value of each *X* line to highlight only relative intensity variations along *X*. The final ACF for a given zone (inside DNA damage or outside DNA damage) is calculated as the average of the ACFs of the selected segments and exported.

The ACFs are analyzed in Origin (OriginLab, Northampton, Massachusetts) using a model that describes only diffusion:

(2a) $$\begin{array}{c} G\left(\tau \right)=G\left(\infty \right)+G\frac{1}{1+\frac{4D\tau }{w_{0}^{2}}} \end{array}$$

only binding:

(2b) $$\begin{array}{c} G\left(\tau \right)=G\left(\infty \right)+G\;\exp\left(-\frac{\tau }{\tau_{B}}\right) \end{array}$$

or diffusion and binding dynamics:

(2c) $$\begin{array}{c} G\left(\tau \right)=G\left(\infty \right)+G\left[F_{D}\left(\frac{1}{1+\frac{4D\tau }{w_{0}^{2}}}\right)+F_{B}\;\exp\left(-\frac{\tau }{\tau_{B}}\right)\right] \end{array}$$

Here, *G*(∞) is an offset, *G* represents the amplitude of the ACF, *D* is the diffusion coefficient, and *w*_0_ is the 1/e^2^ size of the focal spot in the lateral direction. $F_{D}$ corresponds to the diffusion fraction, and $F_{B}=1-F_{D}$ represents the binding fraction. The use of a 2D model of diffusion is justified by the fact that in our experimental conditions, the axial size of the detection volume *w*_z_ is much larger than the lateral size *w*_0_, *w*_z_ >> *w*_0_ (27).

Regarding intensity time trace analysis, the accumulation of PARP1-RFP at damage sites is examined by fitting the short-term intensity versus time profiles (average intensity in the DNA damage region) using the following model:

(3) $$\begin{array}{c} y=y_{B}+A_{on}\;\left[1-e^{-\frac{t-t_{D}}{\tau_{ACC}}}\right] \end{array}$$

where *y*_*B*_ is the intensity before the induction of DNA damage, *t*_*D*_ corresponds to the end of the induction of DNA damage, *τ*_*ACC*_ is the characteristic accumulation time, and *A*_*on*_ is the amplitude of the curve.

Lastly, the long-term decay of the intensity profile was analyzed using a separate fitting model:

(4) $$\begin{array}{c} y=y_{0}+A_{off}e^{-\frac{t-t_{0}}{\tau_{DEC}}} \end{array}$$

where *y*_*0*_ and *t*_*0*_ represent the starting points of the decay, *τ*_*DEC*_ is the characteristic decay time, and *A*_*off*_ is the amplitude.

## Results and discussion

### Measurement of PARP1-RFP dynamics after induction of DNA damage

**Figure 2:**
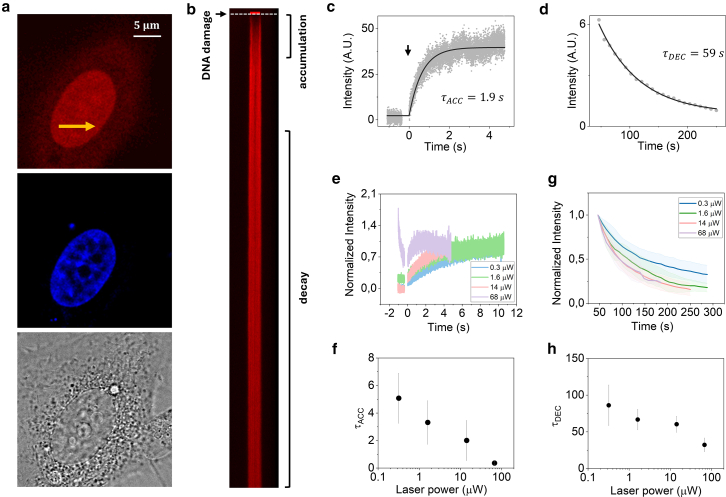
Measurement of PARP1 accumulation dynamics after induction of DNA damage. (*a*) Representative confocal fluorescence (*top* and *middle*) and bright-field (*bottom*) images of HeLa cells transiently transfected with PARP1 chromobody tagged with RFP (PARP1-RFP). Nuclear staining is done with Hoechst. Scale bar: 5 μm (*b*) Fluorescence intensity carpet of the first 120,000 lines of a representative dataset. The white dashed line indicates the end of the laser micro-irradiation event inducing DNA damage. (*c*) Short-term analysis from which we extract a value of $\tau_{ACC}=1.9\;s$. (*d*) Long-term analysis from which we extract a decay time $\tau_{DEC}=59\;s$. (*e*) Short-term intensity time traces at different 561-nm laser powers. Data represent average ± SD of measurements performed on at least 5 cells. (*f*) Average value of $\tau_{ACC}$ extracted from data acquired at different laser powers. (*g*) Long-term intensity time traces at different 561-nm laser powers. Data represent average ± SD of measurements performed on at least 5 cells. (*h*) Average value of $\tau_{DEC}$ extracted from data acquired at different laser powers.

We performed all measurements in the nucleoplasm of HeLa cells expressing a PARP1 chromobody tagged with TagRFP (PARP1-RFP) in regions far from the nucleolus. Hoechst intensity is used as a quantitative reference for nuclear DNA concentration (Fig. 2 *a*). The measurement is performed by fast scanning along the *X* axis over time. After 1024 lines, DNA damage is induced using a 405-nm laser for 512 lines, corresponding to a time of 0.36 s, as shown in Fig. 2 *b*.

Firstly, we focused on the intensity profiles, analyzing the temporal dynamics of PARP1-RFP accumulation and decay, which provide insights into the protein’s response to DNA damage. Fig. 2, *c* and *d*, shows the time-resolved analysis of a typical line scanning dataset acquired using a 561-nm laser with 14 *μ*W of laser power, showing a rapid accumulation and a slow decrease of the intensity. By applying Eqs. 3 and 4, we determine the time constant *τ*_*ACC*_ corresponding to rapid accumulation of PARP1-RFP on the DNA damage region (Fig. 2 *c*) and the time constant *τ*_*DEC*_ corresponding to the slow decay of the intensity (Fig. 2 *d*). We find $\tau_{ACC}=2.1\pm 1.5\;s$ and $\tau_{DEC}=60\pm 11\;s$ (mean ± SD of *n* = 9 cells from 2 independent experiments). The measured value of *τ*_*DEC*_ is shorter compared to the reported times for PARP1’s transient association at DNA damage sites (20,28). In this respect, we note that the value *τ*_*DEC*_ is not only related to the dissociation of PARP1-RFP from damaged sites (required to facilitate PARylation processes) but can also be influenced by motion of the DNA damage region (Fig. S1) and by photobleaching.

To specifically evaluate the potential impact of photobleaching on the measured dynamics, we compared results from data acquired at different excitation laser power (0.3, 1.6, 14, and 68 *μ*W) (Fig. 2, *e*–*h*). Intensity trace analysis revealed that the excitation power affects both the short-term dynamics (Fig. 2, *e* and *f*) and the long-term decay (Fig. 2, *g* and *h*). At the lowest excitation power, the accumulation time of PARP1-RFP is $\tau_{ACC}=5.1\pm 1.8\;s$ (*n* = 20 cells, from 2 independent experiments), in keeping with previous works (20,28) and two times larger compared to the value obtained at 14 *μ*W laser power (Fig. 2, *e* and *f*). For the long-term decay, we obtain $\tau_{DEC}=90\pm 28\;s$ (*n* = 20 cells, from 2 independent experiments) at the lowest laser power condition, but this value decreases with increasing excitation power (Fig. 2, *g* and *h*). We conclude that data acquired at increasing laser power show increased photobleaching, which results in a faster intensity decay and an apparently faster accumulation of PARP1-RFP.

### Measurement of PARP1-RFP mobility at DNA damage sites by segmented FCS

**Figure 3:**
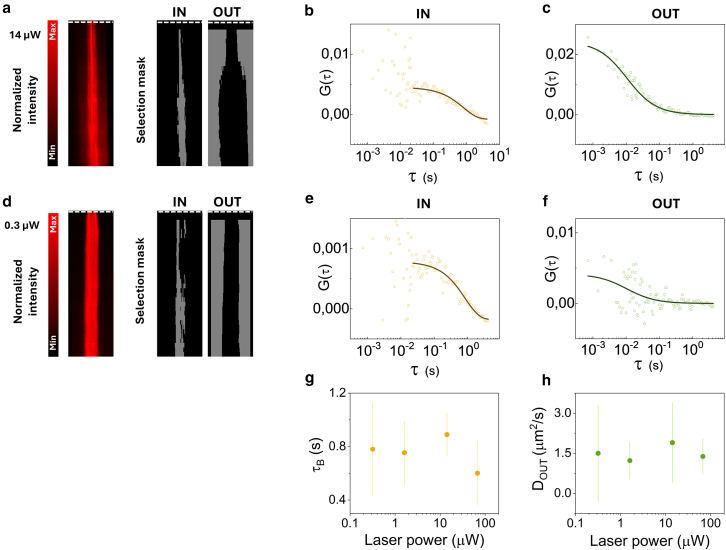
Measurement of the PARP1 mobility at DNA damage sites by segmented FCS. (*a*–*c*) Representative segmented FCS analysis of PARP1-RFP after DNA damage induction for data acquired at 14 *μ*W laser power using a one-component model. (*a*) Normalized segment intensity map and corresponding segment selection map. The white dashed line indicates the end of the laser micro-irradiation event inducing DNA damage. Regions of interest including multiple segments are selected either inside (IN) or outside (OUT) the DNA damage region, based on the value of normalized fluorescence intensity of PARP1-RFP. (*b*) The average ACF corresponding to the DNA damage region is fitted to a one-component binding model (Eq. 2b), restricting the fitting range to lag times *τ* > 20 ms. (*c*) The average ACF outside the DNA damage region is fitted to a one-component diffusion model (Eq. 2a). (*d*–*f*) Representative segmented FCS analysis of PARP1-RFP after DNA damage induction for data acquired at 0.3 *μ*W laser power using a one-component model. (*d*) Normalized segment intensity map and corresponding segment selection map. The white dashed line indicates the end of the laser micro-irradiation event inducing DNA damage. Regions of interest including multiple segments are selected either inside (IN) or outside (OUT) the DNA damage region, based on the value of normalized fluorescence intensity of PARP1-RFP. (*e*) The average ACF corresponding to the DNA damage region is fitted to a one-component binding model (Eq. 2b), restricting the fitting range to lag times *τ* > 20 ms. (*f*) The average ACF outside the DNA damage region is fitted to a one-component diffusion model (Eq. 2a). (*g*) Binding time of PARP1-RFP in the DNA damage region extracted from segmented FCS analysis at different excitation powers. Data represent average ± SD of measurements performed on at least 5 cells. (*h*) Diffusion coefficient of PARP1-RFP outside the DNA damage region extracted from segmented FCS analysis at different excitation powers. Data represent average ± SD of measurements performed on at least 5 cells.

Then, we applied segmented FCS to detect differences in PARP1-RFP diffusion between inside and outside the DNA damage region. Here, temporal segmentation is useful because it can reduce artifacts due to photobleaching or movement of the DNA damage region that deforms the ACF, similar to other reported correction methods (8,29). The effect of the temporal duration T of the segment on the ACF is shown in Fig. S2 for a representative dataset. For the selected segment duration T = 9.2 s, we note that the ACF in the DNA damage region can be described by a fast (diffusive) and a slow (binding) component. If the segment duration is set to 55.8 s, the ACF is dominated by photobleaching or motion. If the segment is set to 0.9 s, the binding component of the ACF is filtered out. For each dataset, regions of interest including multiple segments are selected either inside (Fig. 3 *a*, IN) or outside (Fig. 3 *a*, OUT) the DNA damage region, based on the value of normalized fluorescence intensity. The corresponding average ACFs are fitted using either a two-component model including diffusion and binding (Eq. 2c) (Fig. S3) or a one-component model (only diffusion, Eq. 2a, or only binding, Eq. 2b) (Fig. 3, *b* and *c*).

The two-component analysis of data acquired at 14 *μ*W revealed that, inside the DNA damage region, there is a larger binding fraction ($F_{B}=0.62\pm 0.12$, mean ± SD of *n* = 9 cells from 2 independent experiments) compared to the nondamaged region ($F_{B}=0.17\pm 0.12$, mean ± SD of *n* = 9 cells from 2 independent experiments) (Fig. S3). The large fraction of the diffusion component outside the damaged region indicates a mainly diffusive behavior of PARP1-RFP in these regions**.** Inside the DNA damage region, the average diffusion coefficient of PARP1-RFP was $D=\left(1\pm 1\right)\;\mu m^{2}/s$ with a binding time of $\tau_{B}=\left(1.6\pm 1.8\right)\;s$, whereas outside the DNA damage region, the average value of the diffusion coefficient was $D=\left(3.8\pm 3.1\right)\;\mu m^{2}/s$ (mean ± SD of *n* = 9 cells from 2 independent experiments)**.**

For the data acquired at lower laser power (0.3 *μ*W), as expected, the ACFs are significantly noisier (Figs. 3, *e* and *f*, and S3, *e* and *f*). For this reason, we simplified the model for fitting the data to a single-component model. In this approach, for data inside the damaged region, we considered only the binding component (Eq. 2b) and restricted the fitting range to lag times *τ* > 20 ms (Fig. 3 *e*). For data outside the damage region, we considered only the diffusion component (Eq. 2a; Fig. 3 *f*). This simplified approach allowed us to extract the binding time in the DNA damage region *τ*_*B*_ and the diffusion coefficient outside the DNA damage region, *D*_*OUT*_, as a function of the excitation power (Fig. 3, *g* and *h*). We found that at the lowest excitation power, $\tau_{B}=0.8\pm \;0.3\;s$ and $D_{OUT}=1.5\pm 1.8\;\mu m^{2}/s$ (mean ± SD of *n* = 20 cells from 2 independent experiments) (Fig. 3, *g* and *h*).

Notably, the diffusion coefficient *D*_*OUT*_ of PARP1-RFP extracted from this analysis shows a value comparable to the value of PARP1-RFP in the nucleoplasm of HeLa cells reported in previous studies conducted in the absence of DNA damage (26). This indicates that the diffusion of PARP1 is not altered outside the DNA damage region. The value of binding time *τ*_*B*_ appears to be largely independent from the excitation power, suggesting that it is not affected by the presence of photobleaching, in contrast to the accumulation and decay dynamics (Fig. 2, *e*–*h*). This is due to the temporal segmentation of the data, which reduces the impact of photobleaching on the ACF curves. We note that whereas *τ*_*B*_ represents the average binding time of PARP1 in the DNA damage region (measured using the fluctuations of intensity in the confocal detection volume), the characteristic times *τ*_*ACC*_ and *τ*_*DEC*_ are related to the transient accumulation of PARP1 in the region and may depend on other parameters (e.g., the size and shape of the lesion and the diffusion coefficient).

## Conclusions

In this study, we have demonstrated that segmented FCS can be applied to analyze the recruitment dynamics of PARP1-RFP after induction of DNA damage, highlighting the importance of the FCS technique for studying the recruitment dynamics of proteins involved in DNA damage response mechanisms. Our analysis of samples excited with higher laser power has shown the presence of photobleaching effects, which affect only part of our analysis. The ability to segment data has been crucial in this study because data segmentation allowed us to precisely analyze the dynamics of PARP1-RFP in the region where we induce DNA damage and in regions immediately adjacent to the DNA lesion site. We believe that our approach, based on the use of line scanning in a commercial microscope, could be easily applied to the study of other molecules involved in the DNA damage response.

In summary, our approach allowed us to quantify the mobility in regions inside and outside the DNA damage site, providing a detailed characterization of PARP1 mobility in response to DNA damage. This methodology will be useful to better understand the complex mechanisms of the cellular response to DNA damage in future studies.

## Acknowledgments

The research leading to these results has received funding from 10.13039/501100005010Associazione Italiana per la Ricerca sul Cancro (AIRC) under MFAG (My First AIRC Grant) 2018 – ID 21931 – principal investigator: L.L. Work was supported in part by PRIN-PNRR 2022 project “Liquid-Liquid Phase Separation dynamics in biomimetic compartments” (LLIPS) (project code: P20228CCLL). This work was supported by the 10.13039/501100004505University of Catania under the program Programma Ricerca di Ateneo PIA.CE.RI. 2024–2026 Linea 1 “NANO-STRENGTH” and Linea Open Access. This work has been partially funded by the 10.13039/501100000780European Union (NextGeneration EU) through the MUR-PNRR project SAMOTHRACE (ECS00000022). The work has been partially funded by the National Plan for NRRP Complementary Investments (PNC, established with the decree-law 6 May 2021, no. 59, converted by law no. 101 of 2021) in the call for the funding of research initiatives for technologies and innovative trajectories in the health and care sectors (directorial decree no. 931 of 06-06-2022) — project no. PNC0000003 — Advanced Technologies for Human-Centred Medicine (ANTHEM). This work was supported in part by the 10.13039/501100003196Italian Ministry of Health, Piano di Sviluppo e Coesione del Ministero della Salute 2014–2020, project: Pharma-HUB - Hub per il riposizionamento di farmaci nelle malattie rare del sistema nervoso in età pediatrica (CUP E63C22001680001 - ID T4-AN-04). The authors gratefully acknowledge the Bio-Nanotech Research and Innovation Tower (BRIT; PON project financed by the 10.13039/501100003407Italian Ministry for Education, University and Research [MIUR]). We also acknowledge the national project Minibeam Radiotherapy (MIRO) financed by the National Scientific Committee 5 (CSN5) of the INFN.

## Author contributions

L.L. and A.D. designed the study. E.L. and G.P. prepared the samples. L.L. and E.L. collected the data. L.L. wrote the software. E.L. and L.L. performed data analysis. L.L., A.D., and E.L. discussed the results. L.L. and E.L. wrote the manuscript. All authors critically reviewed the manuscript.

## Declaration of interests

The authors declare no competing interests.

## Supporting material

Document S1. Figures S1–S3

Document S2. Article plus supporting material
