# Supplementary material for: Measuring PARP1 mobility at DNA damage sites by segmented fluorescence correlation spectroscopy
Source: Biophys J. 2025 May 16;124(20):3441–7. doi: 10.1016/j.bpj.2025.05.013 (PMC12709243; doi:10.1016/j.bpj.2025.05.013)
Supplement: Document S1. Figures S1–S3 [file mmc1.pdf]

Biophysical Journal, Volume *124*

Supplemental information

Measuring PARP1 mobility at DNA damage sites by segmented fluorescence correlation spectroscopy

Elisa Longo, Greta Paternò, Alberto Diaspro, and Luca Lanzanò

Supporting information for:

Measuring PARP1 mobility at DNA damage sites by Segmented Fluorescence Correlation Spectroscopy (FCS)

Elisa Longo[1], Greta Paternò[1], Alberto Diaspro[2,3], Luca Lanzanò [1,2,4,5]

[1]Department of Physics and Astronomy "Ettore Majorana", University of Catania, Catania, Italy; [2]Nanoscopy, CHT Erzelli, Istituto Italiano di Tecnologia, Genoa, Italy; [3]DIFILAB, Department of Physics, University of Genoa, Genoa, Italy; [4] Istituto Nazionale di Fisica Nucleare (INFN), Sezione di Catania, Catania, Italy; [5] Centro Siciliano di Fisica Nucleare e Struttura della Materia (CSFNSM), Catania, Italy

Corresponding author:

Luca Lanzanò
Department of Physics and Astronomy "Ettore Majorana", University of Catania

Via S. Sofia, 64 - 95123 Catania (Italy)
Email: luca.lanzano@unict.it
Phone: +39 095 3785330

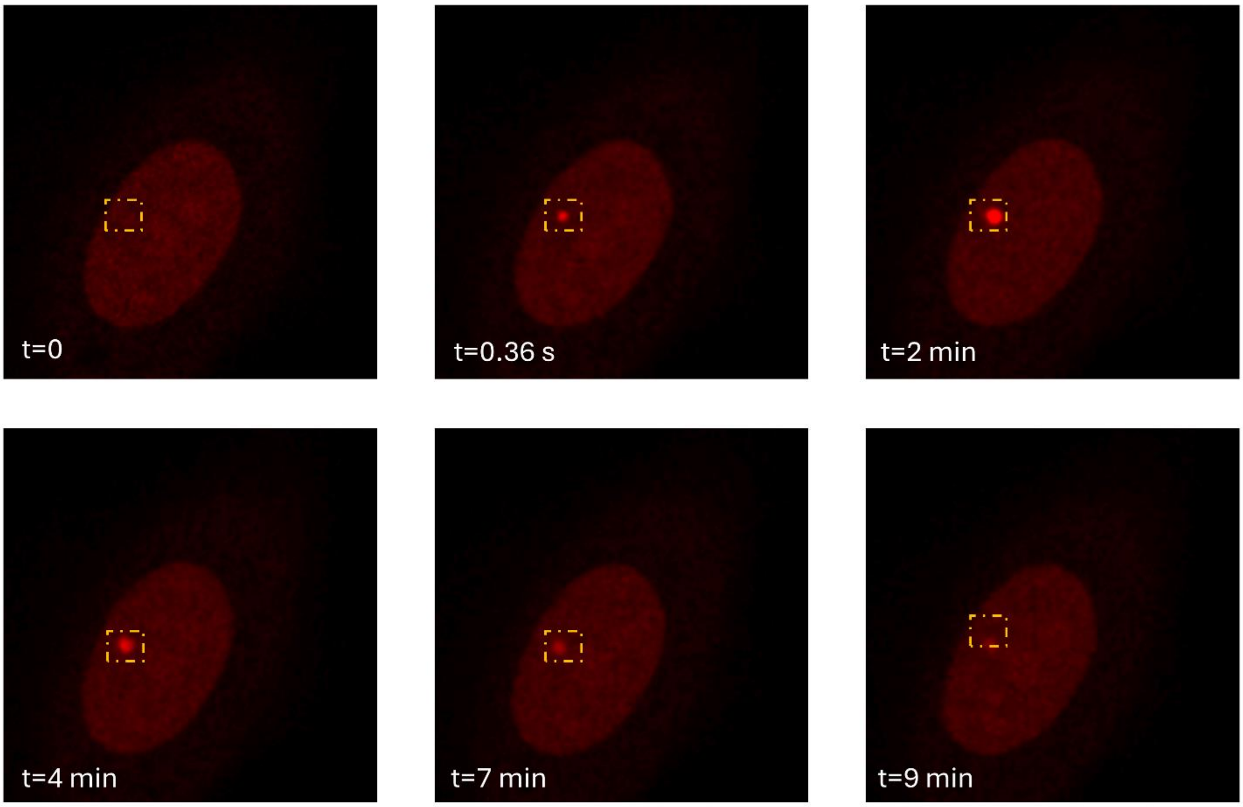

Fig. S1. Time lapse of Hela cells after DNA damage

Time lapse of HeLa cells expressing a PARP1 chromobody tagged with RFP and labelled with Hoechst before (t=0) and after the induction of DNA damage on a small region (0.7µm ×0.7µm) at the center of the yellow square.

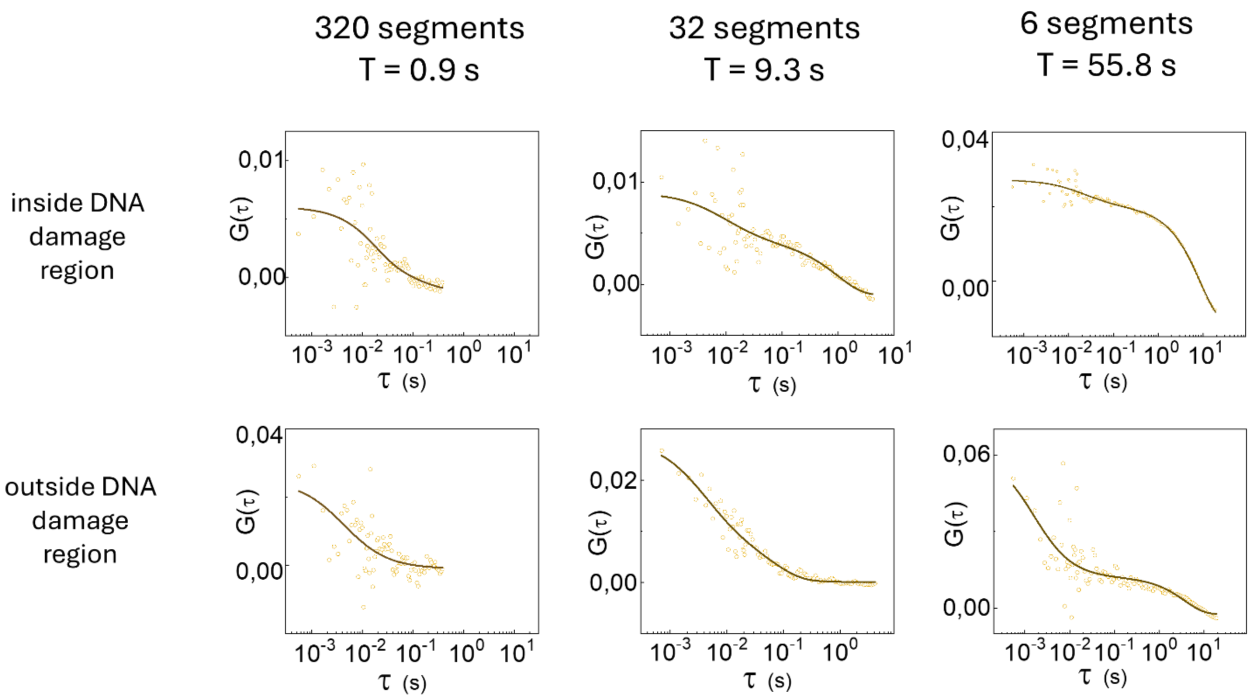

Fig. S2. Segmented FCS of PARP1-RFP at different segment duration

Segmented FCS analysis of a representative dataset for different values of duration of the segment. The plots represent the average ACFs calculated inside the DNA damage region (top) or outside the DNA damage region (bottom) for segment durations corresponding to T=0.9 s, 9.3 s, 55.8 s. The ACFs are fitted to a 2-components model (Eq.2c).

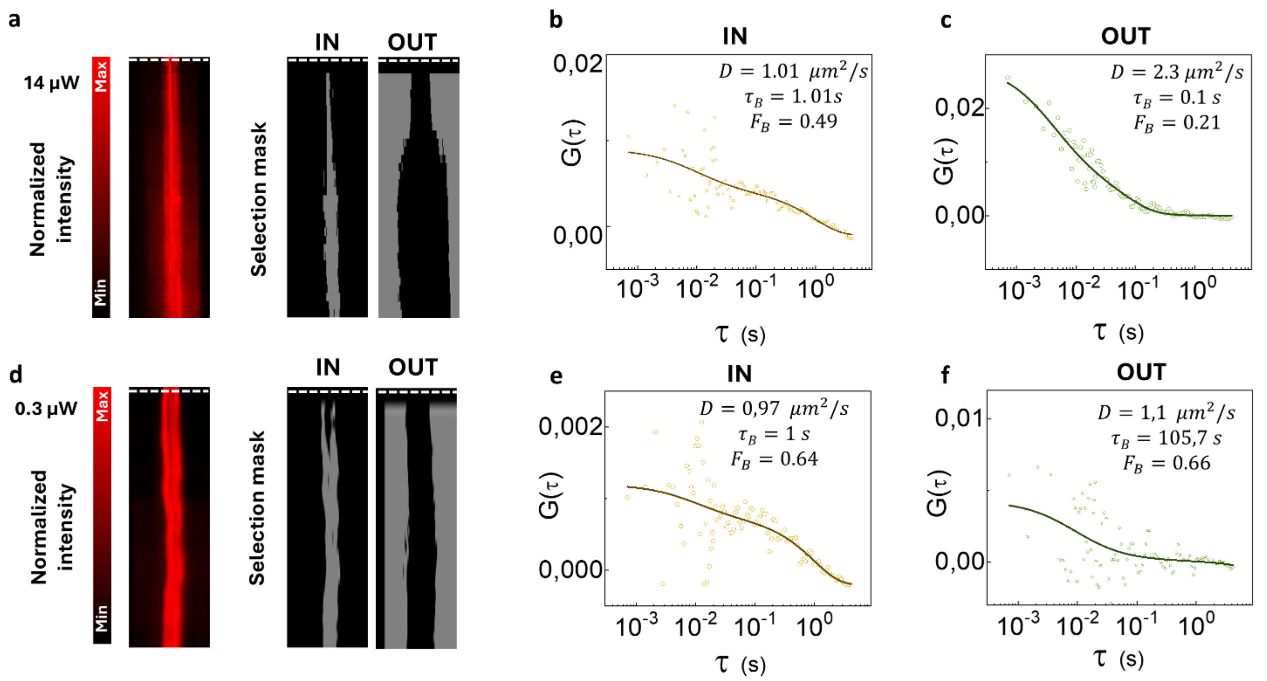

Fig. S3. Measurement of the PARP1 mobility at DNA damage sites by Segmented FCS and fitting using a 2-component model

(a-c) Representative segmented FCS analysis of PARP1-RFP after DNA damage induction for data acquired at 14 µW laser power using a 2-component model. (a) Normalized segment intensity map and corresponding segment selection map. The white dashed line indicates the end of the laser micro-irradiation event inducing DNA damage. Regions of interest including multiple segments are selected either inside (IN) or outside (OUT) the DNA damage region, based on the value of normalized fluorescence intensity of PARP1-RFP. (b,c) The average ACFs corresponding to the DNA damage region (b) or outside the DNA damage region (c) are fitted to a 2-component model including diffusion and binding (Eq.2c).

(d-f) Representative segmented FCS analysis of PARP1-RFP after DNA damage induction for data acquired at 0.3 µW laser power using a 2-component model. (d) Normalized segment intensity map and corresponding segment selection map. The white dashed line indicates the end of the laser micro-irradiation event inducing DNA damage. Regions of interest including multiple segments are selected either inside (IN) or outside (OUT) the DNA damage region, based on the value of normalized fluorescence intensity of PARP1-RFP. (e,f) The average ACFs corresponding to the DNA damage region (e) or outside the DNA damage region (f) are fitted to a 2-component model including diffusion and binding (Eq.2c).
